# Large-scale modeled contemporary and future water temperature estimates for 10774 Midwestern U.S. Lakes

**DOI:** 10.1038/sdata.2017.53

**Published:** 2017-04-25

**Authors:** Luke A. Winslow, Gretchen J.A. Hansen, Jordan S Read, Michael Notaro

**Affiliations:** 1 U.S. Geological Survey, Office of Water Information, Middleton, Wisconsin 53562, USA; 2 Department of Biological Sciences, Rensselaer Polytechnic Institute, Troy, New York 12180, USA; 3 Minnesota Department of Natural Resources, St Paul, Minnesota 55155, USA; 4 University of Wisconsin—Madison, Nelson Institute Center for Climatic Research, Madison, Wisconsin 53706, USA

**Keywords:** Limnology, Freshwater ecology

## Abstract

Climate change has already influenced lake temperatures globally, but understanding future change is challenging. The response of lakes to changing climate drivers is complex due to the nature of lake-atmosphere coupling, ice cover, and stratification. To better understand the diversity of lake responses to climate change and give managers insight on individual lakes, we modelled daily water temperature profiles for 10,774 lakes in Michigan, Minnesota, and Wisconsin for contemporary (1979–2015) and future (2020–2040 and 2080–2100) time periods with climate models based on the Representative Concentration Pathway 8.5, the worst-case emission scenario. In addition to lake-specific daily simulated temperatures, we derived commonly used, ecologically relevant annual metrics of thermal conditions for each lake. We include all supporting lake-specific model parameters, meteorological drivers, and archived code for the model and derived metric calculations. This unique dataset offers landscape-level insight into the impact of climate change on lakes.

## Background & Summary

Understanding how lake ecosystems are affected by climate change is challenging. The response of lakes to changing climate drivers is not always straightforward due to the complex nature of lake-atmosphere coupling and stratification^1,2^. Lakes have highly diverse characteristics that make individual lake climate response difficult to predict. Attributes such as water clarity^3^, lake size and shape^4^, surrounding canopy cover^5^, and depth^6^ all affect how lakes gain, lose, and distribute thermal energy. These attributes interact in complex and difficult to predict ways to determine how a lake responds to changes to meteorological drivers^1^. Using all information available when evaluating how lakes will respond individually is important to understanding and managing for climate change in lakes. Furthermore, understanding the response of biota to changes in seasonal dynamics of stratification and temperature is challenged by complex behavior and life histories that are influenced by multiple temperature cues^7–9^. To fully evaluate how climate change might affect lakes, the full annual dynamics of water temperature are required.

Most previous research projecting lake temperature trends has focused on annual or seasonal averages^1,10,11^. Furthermore, at large scales, researchers have typically relied on statistical models^12^ or lake ‘archetypes’^13–15^ to reduce the large computational and metadata requirements when examining large numbers of lakes across a continuum of key attributes. In contrast, this dataset focuses on real lakes and daily estimated temperature profiles, based on site- and lake-specific data, allowing for detailed examination of climate change effects on specific temperature metrics relevant to the biological and biogeochemical functioning of lakes.

Here we present a database of daily simulated water temperature profiles in 10,774 midwestern United States lakes for contemporary (1979–2015) and two future periods (2020–2039 and 2080–2099). This database includes basic lake-specific attributes relevant to stratification and temperature trends where available. These include lake location and morphological information, water clarity estimates, surrounding canopy height, and all in-situ observations of water temperature.

The motivation for this simulated dataset was to address several questions relevant to managing thousands of lakes in the midwestern United States in a changing climate. For example, (1) How diverse is the response to climate change of a regional population of lakes? (2) What does this diverse response to climate change mean for inland fisheries? (3) What does climate change mean for broad-scale processes, such as nutrient cycling? This novel modeling approach can help fill the gaps in past water temperature data as well as project a scenario of future changes to improve our understanding of the impacts of climate change on lakes and their biota.

## Methods

Modeling contemporary and future water temperatures across Michigan, Minnesota, and Wisconsin requires integrating diverse methods, models and datasets. Preliminary methods used to model lake temperatures for 2,368 lakes in Wisconsin, USA were described in brief in previous publications^16,17^. Here we document the entire modeling process, highlighting improvements and updates to the modeling approach.

**Figure 1:**
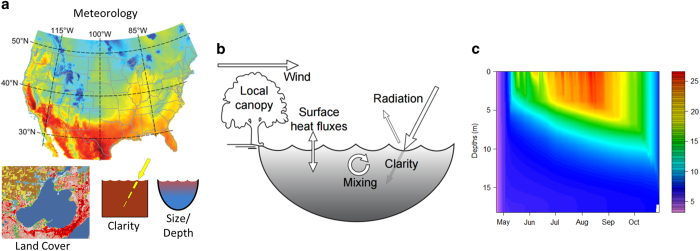
Large-scale lake modeling and metric calculation pipeline. Steps include (**a**) processed meteorological drivers and lake attributes linked to lakes. (**b**) General Lake Model v2.2 model run using lake meteorology and lake attributes to parameterize model and (**c**) daily temperature profiles and calculated thermal metrics archived.

The modeling framework (Fig. 1) consisted of (1) the collation of required and optional lake-specific characteristics across the modeled region and identification of lakes with sufficient data, (2) setup and run of the 1-dimensional hydrodynamic model, and (3) output of raw daily simulated water temperature, and calculation of annual, ecologically relevant thermal metrics.

### Hydrodynamic model

At the core of the modeling framework is a lake hydrodynamic model that uses inputs of lake-specific properties and local meteorology to estimate water temperature. Our chosen model is the open source, General Lake Model (GLM) version 2.2.0 (ref. 18). GLM is a one-dimensional dynamical model which simplifies lakes using a vertical approximation, where horizontal variability is ignored. GLM uses a Lagrangian approach to layer structure where layers can split and combine based on changing vertical gradients. GLM is a modern, implementation of other one-dimensional models^19,20^.

The water budget of GLM can include surface and groundwater inflows/outflows as well as precipitation and evaporation. In our simulations, evaporation is an important energy flux and is included. Precipitation is also included as this replaces water lost to evaporation. We did not include surface or groundwater flows as they were not generally available across the 10,774 lakes and were not a significant contributor to the energy budget of most lakes^16^. Despite the generally small impact of surface inflows on the energy budget^16^, in some lakes, the imbalance of precipitation and evaporation and a lack of compensatory surface or groundwater flows resulted in a long-term negative trend in lake volume. To counteract this while minimizing the impact on temperatures, across all lakes we increased rainfall volume by 17 cm per month during the summer months (July, August, and September). Excess water volume input through precipitation was simply lost to generic overflow (which does not induce mixing) when the lake height exceeds the crest height. This maintained reasonable lake volumes for all simulation periods.

In GLM, Energy lost and gained through the surface layer of the lake models is tabulated for each timestep and converted to temperature increases or decreases. Surface energy fluxes include shortwave radiation, longwave radiation, and sensible and latent heat fluxes. Incoming shortwave and longwave radiation are time series inputs to the model, while the outgoing longwave radiation flux is calculated as a function of surface temperature. Sensible and latent heat fluxes are estimated by GLM as a function of surface temperature and the time series drivers of air temperature, relative humidity, and windspeed^18^. For each timestep, these fluxes are summed and result in lowering or increasing the surface water temperature (or ice formation). Ice formation, loss, and snow is modeled using the same structure as other existing 1-dimensional hydrodynamic models, a three-layer system of white ice, blue ice, and snow^21,22^.

Temperatures in the layers below the uppermost surface layer are altered for each time step according to the amount of vertical mixing generated by wind, convection, or inflows; the model also estimates warming of the water column contributed by the attenuation of penetrating shortwave radiation. For our modeling efforts, surface flows were ignored, so all mixing energy originated from wind energy or convective heat loss. The momentum flux from wind is partitioned by GLM into three fates for the kinetic mixing energy, and each is parameterized separately. This energy is converted into wind stirring, wind shear, and Kelvin-Helmholtz billows, and the efficiency of these energetic fates are parameterized user-defined constants (Table 1). As in a previous work^16^, we scaled the momentum flux (impacting the magnitude of all three) according to sheltering from a local canopy buffer (see *Model Parameter* sub-section). Convective mixing is quantified by the turbulent velocity scale of convective cooling (w*^23^) calculated for the GLM lagrangian model following (ref. 19). The contribution of light attenuation to water column warming is calculated according to the Beer-Lambert Law, which parametrizes an exponential attenuation of energy with increasing depth^24^. Attenuated solar radiation is converted into thermal energy, and thus a temperature increase.

### Meteorological data

Meteorological data used to drive the lake simulations have two primary sources, the North American Land Data Assimilation^25^ (NLDAS; http://ldas.gsfc.nasa.gov/nldas) and downscaled Global Climate Models (GCMs). NLDAS was used to drive back-cast simulations (1979–2015). To represent future climate conditions under anthropogenic climate change, we used six regionally downscaled GCMs from the Coupled Model Intercomparison Project Phase 5 (CMIP5). Of the GCMs available in the CMIP5 archive, we selected a subset of models that met three requirements. (1) sufficient spatial resolution for the downscaling procedure, (2) satisfactory representation of the Great Lakes Basin’s modern day climatology, and (3) represent a range of responses to show the diversity of modeled outcomes^26,27^ (further discussion of GCM selection by (ref. 27)). The six GCMs downscaled and used for our simulations were ACCESS, CNRM, GFDL, IPSL, MIROC5, and MRI (Table 2). All GMCs were driven by the representative concentration pathway 8.5 (RCP8.5), which assumes high warming in response to continued growth in emissions through the end of the 21st century.

The six GCMs from CMIP5 were downscaled from the native model resolution (various) to a common horizontal 25-km grid spacing and debiased to local conditions for the overlapping 1981–2000 period. Downscaling was done using the International Centre for Theoretical Physics (ICTP) Regional Climate Model Version Four^28,29^ (RegCM4). Debiasing was done against observational data on air temperature, precipitation, near-surface wind speed, relative humidity, and incoming longwave and shortwave radiation using the following observational datasets: Oak Ridge National Laboratory’s Daymet^30^ and the Global Land Data Assimilation System (Version One, GLDAS; http://ldas.gsfc.nasa.gov/gldas/). Debiasing corrected for biases in daily mean and interannual standard deviation for each calendar day. For each day’s simulated value, the climatological mean was subtracted. The remainder was multiplied by the ratio of observed interannual standard deviation to the simulated interannual standard deviation for that calendar day. Finally, the observed climatology for that calendar mean was added to each day’s value. Further details provided by (ref. 31).

Neither the downscaled future GCMs nor NLDAS included precipitation differentiated between snow and rain, instead including water equivalent precipitation for all seasons. For modeling winter ice cover, it is important to differentiate between snow and rain because snow accumulation on ice acts to reduce heat flux through increased albedo and insulation of sensible heat flux, whereas rain tends to melt or compact snow, reducing snow albedo and its insulating effect. To separate water equivalent precipitation into rain and snow for input into the lake models, we used a simple 0 °C air temperature cutoff. While air temperature was above and equal to 0 °C, all precipitation was assumed to be rain. For air temperature below 0 °C, precipitation was assumed to be snow and converted to snow. The magnitude of snowfall was based on a 1:10 conversion (1 cm rainfall equivalent to 10 cm snowfall), assuming a snow density of approximately 100 kg m^−3^.

To drive the one-dimensional hydrodynamic model, NLDAS and the downscaled GCMs were spatially subset to extract local, lake-specific drivers. For all lakes, the lake polygon centroid was used to extract data from the nearest grid cell of the gridded drivers. Due to the large size of the gridded driver datasets, subsets were extracted from remote servers hosting the data using the Geo Data Portal^32^ access from R using the package *geoknife*^33^. Beyond the conversion to snow, all subset NLDAS and GCM data were used without modification with the exception of wind speed data from NLDAS. Wind speeds in the study region showed a small step change in average annual magnitude before and after December 31st, 2001 that is attributed to a switch from back-casting to now-casting of the NLDAS system. To compensate for this, all wind speed values after December 31, 2001 were multiplied by a correction factor (0.921) calculated from a comparison of the speed distributions before and after the 2001 step change.

### Model parameters

Estimating how lake-surrounding land cover (e.g., tall tree canopy) acts to shelter a lake from wind is important in lake hydrodynamic models^4,5^. Wind plays an important role in quantifying surface heat flux and in estimating the mixing dynamics of wind-driven turbulence (details above in modeling section). For our simulations, we used a wind sheltering model based on the lake-surrounding canopy height^5^. To estimate canopy height, we used an approach that uses the land-cover in a 100 meter buffer around each lake previously shown to estimate lake wind sheltering^34^. For the land cover source, we used the U.S. National Land Cover Database^35^ (NLCD 2011) to identify the dominant surrounding land-cover type for each lake. The dominant land-cover was used to select an average canopy height for each type using the lookup table^34^ (Supplementary Table 1).

For sub-surface lake morphology, GLM is parameterized using lake hypsometry, or the relationship between depth and area. For hypsometry for our lakes, we used a tiered approach. When available, observed lake hypsometry was used to parameterize the model. But for most lakes, hypsometry is not known, or has not been digitized. For those lakes, we used an estimate of maximum depth and assumed a conical hypsometric profile. We required either hypsometry or an observation of maximum depth to run a lake model, and the lack of maximum depth was the most common reason a lake was excluded from this modeling effort.

Average light attenuation (Kd) is an important parameter in estimating lake thermal structure as it controls the vertical distribution of radiative heat flux. To parameterize our model, we used *in situ* Secchi observations from a variety of databases. The majority of data came from the state natural resources departments (the Michigan, Minnesota and Wisconsin Departments of Natural Resources). Other minority providers included, but were not limited to the U.S. Geological Survey, tribal agencies, and local county or municipalities. The observed Secchi depth observation was converted to light attenuation using a constant conversion coefficient of 1.7 (ref. 36). When more than one observation was available for a single lake, we took the annual mean of all Secchi observations before converting to light attenuation. If no *in situ* observations were available, the overall average mean Secchi value (2.46 meters) was used. Of the 10,774 modeled lakes, 5,716 had *in situ* Secchi observations, while the remaining 5,058 use the global mean Secchi.

### Modeled lakes

All lakes modeled as part of this study were selected from the U.S. Geological Survey’s National Hydrography Dataset, specifically the 1:100 k, medium resolution product^37^ (NHD; accessed January 2016). To align with previous large-scale lake data collection efforts^38^ and to reduce the number of candidate lakes to a more manageable number, we adopted a 4 hectare minimum size cutoff (based on lake polygon areas calculated from the NHD). With the cutoff, there were 27,315 individual lakes. Of these, only lakes with available observed maximum depth (or hypsometry) and coverage from gridded driver data (10,774 lakes) were modeled.

### Thermal habitat metrics

Derived thermal metrics were calculated for all simulated lakes to enable easier use of the large simulation output and that may be of use towards answering a variety of limnological questions. These derived outputs are a generic set of metrics that were chosen to be broadly applicable to questions of change in lake thermal structure due to climate change. While the open-source released code is the primary source for details on the calculation methods (Table 3), we have also included the full list of derived outputs (Supplementary Table 2) and briefly discuss the thermal metrics and how they were calculated.

The core metrics include calculated annual metrics of broad interest. Growing degree days with base temperatures of 0, 5, and 10 °C were calculated as a measure of cumulative thermal energy. Ice cover duration was included and defined as the total number of days with ice cover and includes all days in which the lake was ice covered, not just the longest contiguous period. Ice onset date and ice breakup date were calculated and defined as the last open day of the year in the fall/winter and the first open day of the year in the spring. Using the previous ice off date definition, the coefficient of variability (standard deviation divided by the mean) of surface water temperatures for 0 to 30 days after ice-off and for 30 to 60 days after ice-off was calculated.

Common stratification metrics were included for those interested in predicted changes to mixing and stratification^39^. Overall stratification duration was calculated as the number of days in the longest contiguous stratified period, where lakes were considered stratified when the water column temperature difference from the surface to bottom of the lake was equal or greater than 1 °C. Stratification onset date was also calculated as the first modeled day meeting the earlier definition of stratified. Using the first day of stratification as defined earlier, bottom temperature at stratification onset was extracted. The average thermocline depth during the stratified period was calculated using the R package *rLakeAnalyzer*^40^ using the seasonal option to avoid erroneous calculations caused by temporary microstratification. Annual sum of daily Schmidt stability^41^ was calculated.

Lastly, temperatures aggregated by season and month were calculated. For all 12 months, mean and max temperatures were calculated for surface (shallowest simulated layer) and bottom (deepest simulated layer) waters. To facilitate comparison with past empirical work on lake temperature trends^1,42^ we included July through September annual average and maximum modeled surface temperatures for each lake and year.

### Code availability

All code and Supplementary Data for the overarching modelling framework is open-source and freely available in the form of several R packages and a core hydrodynamic model hosted online on Github (Table 3). The source code for the open-source model GLM is available online at Github (https://github.com/AquaticEcoDynamics). The custom packages developed for this effort are *GLMr*, *glmtools*, *lakeattributes*, and *mda.lakes*. *GLMr* and *glmtools* are packages which automate and aid in the creation of model input files and processing of output from GLM. The *mda.lakes* package contains all routines to combine the collected metadata into the appropriate GLM input files and process the resulting output into the various ecologically-relevant thermal metrics. The *lakeattributes* package is a container for key attributes of all modeled lakes uniformly linked by NHD permanent ID (site_id identifier is a combination of ‘NHD_’ prefix and NHD permanent ID) and includes Secchi observations, water temperature observations (for validation), lake latitude/longitude, the land-use within a 100 m buffer, lake surface area, and lake hypsometry (or maximum depth). The collection of code and routines used in preparation of NHD lake data and spatial linking to attribute data is also available online at the *necsc-lake-modeling* Github repository. Snapshots of the source of the custom R packages used to produce these model results have been archived and have version-specific DOI references (Table 3).

## Data Records

The model output data are available as a number of tab-separated text files, zip archive files, and shapefile (Data Citation 1; Table 4). The zip archive files contain the raw water temperature outputs for all lakes as individual comma-separated files. The zip files are split into groups of 1,000 lakes to avoid distributing a single 20 gigabyte zip file. A lookup table is included to map individual lake IDs to the relevant zip archive files. Annual thermal habitat metrics derived from the raw simulation data are also available as a single file with a row for each lake and year. The shapefile includes polygons for all simulated lakes indexed by the same site identifier used throughout the data release.

The dataset output is organized by output type (i.e., raw temperature, derived metrics, and meteorological drivers) and is distributed as different sets of files. The output based on NLDAS includes data from each lake and year from 1979–2015. All GCM-based output includes data from each lake for each year within three time periods 1981–2000, 2040–2059, 2080–2099.

Also included in the data release are packaged input data (Table 4) that include all lake-specific drivers and parameters used in each simulation. All processed meteorological drivers are saved as lake-specific CSV files (the specific CSV format is input-ready for running GLM) and packaged in zip archives, organized by meteorological driver dataset. All raw model input files are combined and archived as a JSON-formatted ASCII file. A shapefile of all modeled lakes has the modeled subset of the NHD and an attached attribute table that includes lake metadata from the NHD source (e.g., lake name, permanent identifier).

## Technical Validation

### Temperature validation

To validate the model, water temperature estimates were compared to *in-situ*, depth-specific temperature observations from scientific and routine monitoring collections. We collated a large number of observations across many lakes and across the modeled contemporary period (1979–2015). The sources of temperature observations were diverse, with the majority coming from state agencies, followed by collections from federal agencies and universities. Access to these data was greatly simplified by using the U.S. Geological Survey’s (USGS) Water Quality Portal, which aggregates access to multiple databases, including the U.S. Environmental Protection Agency (EPA) Storage and Retrieval Data Warehouse (STORET) and USGS National Water Information System (NWIS) databases. Water temperature data from the North Temperate Lakes, Long-Term Ecological Research (NTL-LTER) site were also retrieved and used as part of the validation analysis. In total across the three states, 1.58 million water temperature observations were collated representing 5,103 lakes.

**Figure 2:**
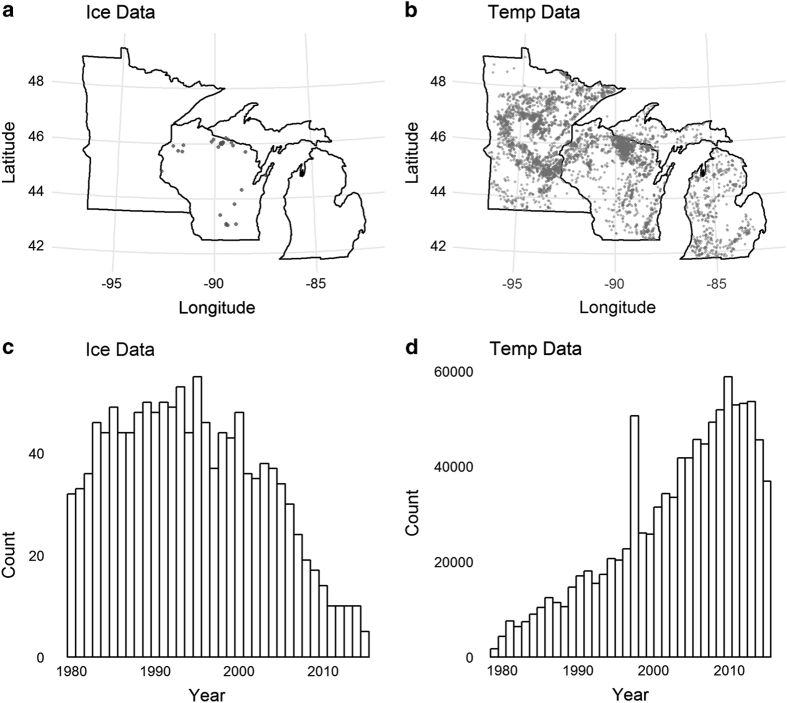
Spatial and temporal distribution of ice and water temperature validation data. Ice observations (**a**,**c**) and water temperature (**b**,**d**) observations used in this study had different temporal and spatial distributions and represent an aggregate across multiple source databases.

Before use in model validation, the data were cleaned of outliers and linked to specific lakes. All temperature data were converted to °C and all outliers<0 °C and >40 °C were removed. Any observations lacking metadata on the depth of observation were also removed. All data from the Water Quality Portal included latitude and longitude information. We used the *sp* package in R^43^ to join all point data representing the observations with the NHD lake polygons using a simple point-in-polygon technique. All temperature observations with points lacking a matching lake polygon were removed. Some observations did not correspond to simulated lakes (due to other missing metadata) so the final validation observations count used was lower than the total observations collated (Fig. 2b,d; 0.94 million matching water temp observations). For consistency and ease of comparison with past work, model error in temperature is reported as the root-mean squared error (RMSE) and error in ice-on and ice-off are reported in both RMSE and Mean Absolute Error (MAE).

**Figure 3:**
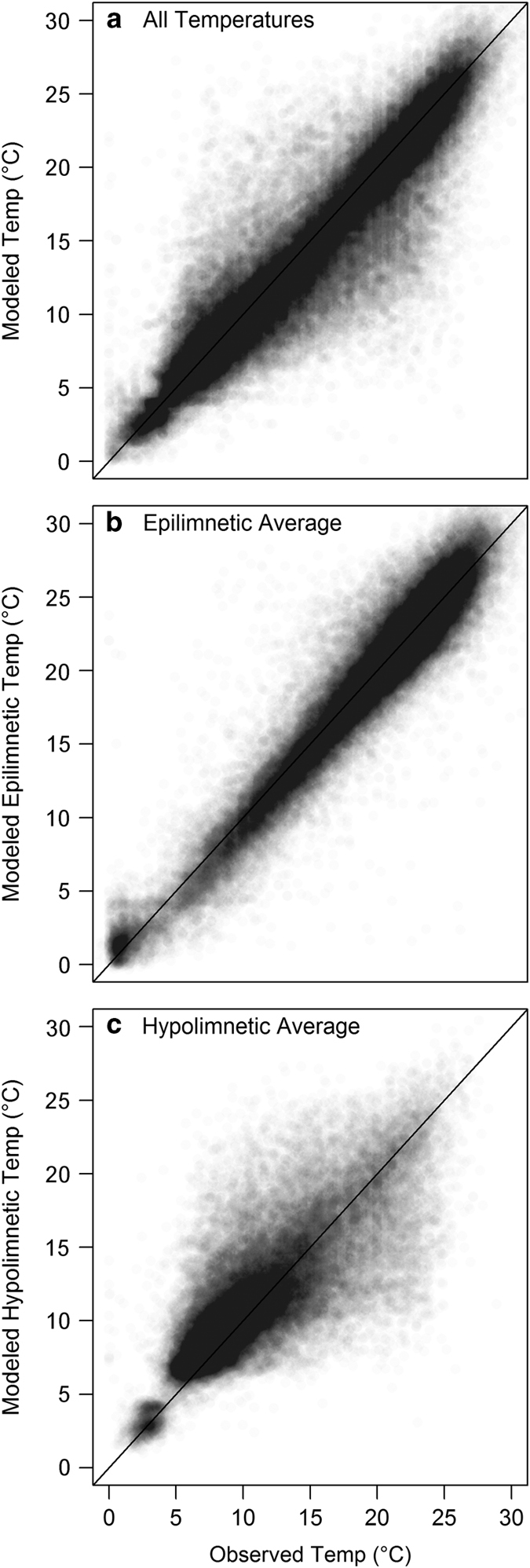
Observed versus modeled temperature for the full three-state validation dataset (3,952 lakes). (**a**) All individual temperature observations (decimated to 10% of full population for improved visualization), (**b**) epilimnetic average temperatures (during stratified periods only) and (**c**) hypolimnetic average temperatures.

All available temperature observations were paired with modeled water temperature from the same lake, depth, and date to estimate model error across lakes (Fig. 3). Overall, simulations reproduced temperature dynamics well, with an overarching all-observation RMSE of 2.79 °C (*n*=936,673). Average epilimnetic modeled temperatures had the lowest residual error when compared to observations, with an average epilimnetic temperature RMSE of 1.91 °C (*n*=72,232). Average hypolimnion temperatures had overall higher error with an RMSE of 3.14 °C (*n*=49,909). For detail and context on error with this large-scale modeling framework, see (ref. 16).

### Ice validation

To evaluate the lake ice model, we compared observations of ice onset and breakup with estimates from the model. Ice onset and breakup observations were not as common as temperature observations, but at least one observation of ice onset or breakup date was available for 29 Wisconsin lakes from the Wisconsin Department of Natural Resources database (Fig. 2a,c; http://dnr.wi.gov/topic/surfacewater/swims/). There was a total of 724 ice onset and 548 ice breakup event observations that overlapped with simulated lakes. Observations were made between 1980 and 2015, though the observation years were biased towards earlier years (Fig. 2c).

**Figure 4:**
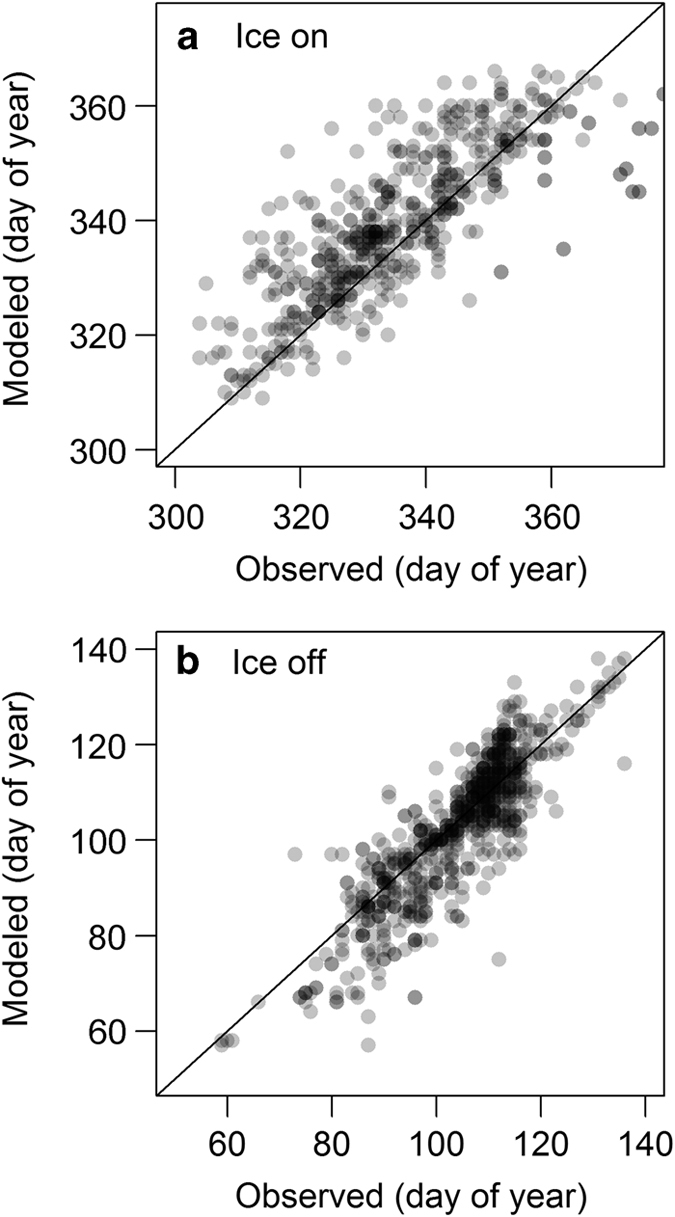
Observed versus modeled ice on and off for all 29 Wisconsin lakes (1,272 observations). Ice onset (**a**) and ice breakup (**b**) dates represented as day of year. Day of year for ice on can be greater than 365 because 365 was added to events that occurred in January or February to create a continuous dataset.

There was large variability in lake ice breakup dates that were well represented by the model (Fig. 4). Lake ice breakup date was more accurately modeled than ice onset date. The RMSE of ice breakup and ice onset were 7.85 days and 12.30 days respectively (MAE of 5.7 and 8.0 days respectively). Bias (modeled—observed) in onset date was also higher, with modeled bias of −2.56 days ice onset. Ice breakup dates bias was lower at 1.57 days later than observed dates. Though only comparable when both ice onset and breakup were observed in the same lake in the same year, RMSE of observed versus modeled ice cover duration was 16.84 days with a 2.03 day shorter modeled ice cover bias. While lake ice models at this scale are not common, past work describing empirical modelling efforts and ice cover estimates based on satellite remote sensing can provide valuable context. For example, an empirical model of ice cover for lakes across Canada have similar error magnitude, with MAE of 8.7 and 5.2 days for ice-on and ice-off respectively^13^. Satellite remote sensing-based estimates of ice cover show similar error magnitude, with studies reporting various observation uncertainties (breakup RMSE values of 7.1 days and MAE of 4.2 days; ice onset MAE of 7.4 days)^44^. We are unaware any reported error in estimated ice cover duration. Overall, the error in modeled ice cover is similar to past broad-scale estimates of lake ice.

## Usage Notes

The downscaled climate drivers represent past and future climate, not specific weather patterns. Future periods are best analyzed as period averages than single-year predictions. For the past overlapping period, the historic weather events will not be represented in the past model results based on the GCM downscaled climate drivers. These overlapping periods are included because they are useful for contrast with future periods and the evaluation of bias in model output that may be a result of the different GCMs. For analyses where the re-creation of specific weather patterns is important (for analysis of past data), use simulations based on gridded past weather (NLDAS), not downscaled GCM simulations.

## Additional Information

**How to cite this article:** Winslow, L. A. *et al.* Large-scale modeled contemporary and future water temperature estimates for 10774 Midwestern U.S. Lakes. *Sci. Data* 4: 170053 doi: 10.1038/sdata.2017.53 (2017).

**Publisher’s note:** Springer Nature remains neutral with regard to jurisdictional claims in published maps and institutional affiliations.

## Supplementary Material



Supplementary Table 1

Supplementary Table 2

## Figures and Tables

**Table 1:** Key parameter values used for GLM simulations of the 10,774 midwestern lakes.

**Parameter**	**Value**
Minimum layer thickness (m)	0.2
Maximum layer thickness (m)	varied; 0.3–1.5
Bulk aerodynamic transport coefficients	0.0013
Convective overturn mixing efficiency	0.125
Wind stirring efficiency	0.23
Shear production efficiency	0.2
Kelvin-Helmholtz turbulent billows mixing efficiency	0.3
Hypolimnetic turbulence mixing efficiency	0.5

**Table 2:** Details on the six selected CMIP5 models used in future simulations of lake temperature conditions.

**Modeling Center (or Group)**	**Institute ID**	**Model Short Name**
Commonwealth Scientific and Industrial Research Organization (CSIRO) and Bureau of Meteorology (BOM), Australia	CSIRO-BOM	ACCESS
Centre National de Recherches Météorologiques/Centre Européen de Recherche et Formation Avancée en Calcul Scientifique	CNRM-CERFACS	CNRM
NOAA Geophysical Fluid Dynamics Laboratory	NOAA GFDL	GFDL
Institut Pierre-Simon Laplace	IPSL	IPSL
Atmosphere and Ocean Research Institute (The University of Tokyo), National Institute for Environmental Studies, and Japan Agency for Marine-Earth Science and Technology	MIROC	MIROC5
Meteorological Research Institute	MRI	MRI

**Table 3:** Citation information for all code collections and custom R packages used in dataset generation.

**R Package/Repository Name**	**Data Generating version (Snapshot DOI)**	**Development Version**
GLMr	10.5281/zenodo.154685	http://github.com/GLEON/GLMr
glmtools	10.5281/zenodo.154689	http://github.com/USGS-R/glmtools
lakeattributes	10.5281/zenodo.154688	http://github.com/USGS-R/lakeattributes
mda.lakes	10.5281/zenodo.154686	http://github.com/USGS-R/mda.lakes
necsc-lake-modeling	10.5281/zenodo.154687	http://github.com/USGS-R/necsc-lake-modeling

**Table 4:** Description of processed model input and output files included in the data release. Where files are differentiated by meteorological data source, files are differentiated by the file name prefix.

**Input/ Output**	**File Name**	**Description**
Output	*DRIVER*_thermal_metrics.tsv	Tab separated file with all generic temperature metrics for all simulated years and lakes. Details included in methods and Supplementary File 1.
Output	*DRIVER*_wtemp_index.tsv	Lookup table for raw water temperature archive files. Links lakes by ID to archive zip file. Useful for extracting raw water temperature for an individual or small number of lakes.
Output	*DRIVER*_wtemp_#.zip	Raw simulated water temperature profile data compressed and archived. Files archived in groups to avoid a single large archive file. ‘#’ character indicates a number from 1 to n, where n is the number of zip files.
Input	model_config.json	JSON (Javascript Object Notation) format file with model configurations for all lakes. Only the meterological driver source and date range modeled varied between meteorological drivers.
Input	model_lakes.zip	Shapefile contained in a zip archive of all polygons for modeled lakes. Attribute table includes the unique identifier for each lake (site_id) to link lake shape to contents of other released output.
Input	*DRIVER*_driver_files.zip	Zip archives of all meteorological drivers used for simulated temperature models. Stored as daily average meteorology in comma-separated files for input to the GLM 1-dimensional model.
*DRIVER*—indicates source of meteorological driver data used to generate data. Is one of processed driver datasets described in methods (Table 1).		
